# Two cases of monkeypox imported to the United Kingdom, September 2018

**DOI:** 10.2807/1560-7917.ES.2018.23.38.1800509

**Published:** 2018-09-20

**Authors:** Aisling Vaughan, Emma Aarons, John Astbury, Sooria Balasegaram, Mike Beadsworth, Charles R Beck, Meera Chand, Catherine O’Connor, Jake Dunning, Sam Ghebrehewet, Nick Harper, Ruth Howlett-Shipley, Chikwe Ihekweazu, Michael Jacobs, Lukeki Kaindama, Parisha Katwa, Saye Khoo, Lucy Lamb, Sharon Mawdsley, Dilys Morgan, Ruth Palmer, Nick Phin, Katherine Russell, Bengü Said, Andrew Simpson, Roberto Vivancos, Michael Wade, Amanda Walsh, Jennifer Wilburn

**Affiliations:** 1 Emerging Infections and Zoonoses Section, National Infection Service, Public Health England, Colindale, London, United Kingdom; 2 Rare and Imported Pathogens Laboratory, Public Health England, Porton, Salisbury, United Kingdom; 3 Field Service, National Infection Service, Public Health England, United Kingdom; 4 Tropical and Infectious Disease Unit, Royal Liverpool University Hospital, Liverpool, United Kingdom; 5 University of Liverpool, Liverpool, United Kingdom, Liverpool, United Kingdom; 6 National Infection Service, Public Health England, Colindale, London, United Kingdom; 7 Guy’s and St Thomas’ NHS Foundation Trust, London, United Kingdom; 8 NIHR Health Protection Research Unit in Respiratory Infections, Imperial College London, London, United Kingdom; 9 Department of Infection, Royal Free London NHS Foundation Trust, London, United Kingdom; 10 Blackpool Teaching Hospitals NHS Foundation Trust, Blackpool, United Kingdom; 11 Defence Medical Services, Ministry of Defence (MOD), United Kingdom; 12 Nigeria Centre for Disease Control (NCDC), Abuja, Nigeria; 13 Travel and Migrant Health Section, National Infection Service, Public Health England, Colindale, London, United Kingdom; 14 Population Health Sciences, Bristol Medical School, University of Bristol, Bristol, United Kingdom; 15 NIHR Health Protection Research Unit in Emerging and Zoonotic Infections, University of Liverpool, Liverpool, United Kingdom; 16 NIHR Health Protection Research Unit in Gastrointestinal Infections, University of Liverpool, Liverpool, United Kingdom

**Keywords:** Monkeypox, United Kingdom, England, Poxvirus, Nigeria

## Abstract

In early September 2018, two cases of monkeypox were reported in the United Kingdom (UK), diagnosed on 7 September in Cornwall (South West England) and 11 September in Blackpool (North West England). The cases were epidemiologically unconnected and had recently travelled to the UK from Nigeria, where monkeypox is currently circulating. We describe the epidemiology and the public health response for the first diagnosed cases outside the African continent since 2003.

Monkeypox is a rare viral zoonotic disease that occurs mostly in Central and West Africa. In this report, we detail the identification of two separately imported cases of monkeypox to the United Kingdom (UK) in September 2018 and the public health response. Each case was managed as a separate incident in the absence of epidemiological evidence linking them in the UK and the public health responses were conducted accordingly.

## Case report 1

The first case, a Nigerian naval officer who was attending a training course at a naval base in Cornwall in the south-west of England, was notified to Public Health England (PHE) on 7 September. He arrived in London from Abuja, Nigeria on 2 September and travelled from London to the military base in Cornwall by train on the same day. The case presented to the general practitioner on the naval base on 3 September with fever, lymphadenopathy and a rash in the groin area that had developed the day before leaving Nigeria. The rash was initially thought to be due to a staphylococcal infection and was treated with antibiotics. On 6 September, the rash had spread to the torso, face and arms and after re-examination the patient was isolated in his quarters. Multiple samples including swabs of the lesions were sent for testing at the PHE Rare and Imported Pathogen Laboratory (RIPL). Monkeypox virus DNA was detected by multiple molecular assays and subsequently confirmed by sequencing analysis. The patient was then transported to the High Consequence Infectious Disease (HCID) Unit at the Royal Free Hospital in London. The clinical condition of the case is stable and he is improving.

## Case report 2

On 10 September, PHE was notified of a second suspected case of monkeypox infection; the diagnosis was confirmed on 11 September. The individual is a UK resident who had returned from a 22-day holiday in Nigeria on 4 September on a flight via Paris, France. He presented to the Accident and Emergency department at Blackpool Teaching Hospitals on 6 September with fever, lymphadenopathy, a scrotal lump and an itchy maculopapular rash. The rash was reported to have started before departing Nigeria on the face and later spread to other areas including the palms of the hands and had become pustular. The patient reported being unwell for approximately one week before presentation, following a different febrile illness that had been treated with antibiotics in Nigeria. On clinical examination the patient had crops of vesicles that were progressing and lesions on the mucosal surfaces of the mouth. The patient was isolated at Blackpool Teaching Hospitals from 7 September and multiple samples, including swabs from the lesions, sent to RIPL confirmed the presence of monkeypox DNA by multiple molecular assays. Although the patient was isolated, monkeypox was not initially suspected because the first lesions appeared in the groin, and the wearing of full personal protective equipment (a filtering face-piece with three indicating levels of protection (FFP3), eye protection, gloves and sterile disposable gown) was not implemented immediately. A number of healthcare workers (HCW) were potentially exposed as a consequence. The case was transferred to the HCID Unit at the Royal Liverpool University Hospital on 10 September where they remain in a stable condition. 

While the source of infection is not yet known, the patient reported contact with an individual with a monkeypox-like rash at a large family event and consumption of bush meat during his visit to a rural area of Nigeria. Since notification of the first case, no other cases have been identified. 

## Public health response

The focus of the public health response in the UK has been to detect possible cases early, mitigate risks and minimise the potential for transmission and secondary cases, as well as to support cases in accessing appropriate clinical management. Ongoing response activities are in collaboration with national and international colleagues, including the Nigeria Centre for Disease Control and other partners.

Although there was no evidence for an epidemiological link between the two cases in the UK, both had travelled in southern Nigeria before coming to the UK. While it was difficult to obtain clear travel histories from the patients, both cases had visited areas in southern Nigeria (Lagos State, Federal Capital Territory, Rivers State and Delta State), where cases of monkeypox have recently been reported (data not shown). The Nigeria Centre for Disease Control is currently conducting epidemiological investigations to identify contacts of these cases and to determine the source of infection.

As part of the response, PHE developed a range of public information and guidance on monkeypox [1] and is liaising with European public health agencies via the Early Warning and Response System (EWRS) as well as other international public health agencies including the European Centres for Disease Prevention and Control (ECDC) and the World Health Organization (WHO) about non-UK contacts and for rapid reporting of public health events.

### Contact tracing

As a precautionary measure, PHE identified possible contacts in the UK to carry out a risk assessment of their contact with the patient and to provide them with advice and information. The first incident was addressed with a contact-based approach to categorise contacts. By the time of the second incident, the use of vaccine was being considered and a risk-based approach was adopted to facilitate this. Although not ideal, in practice this worked because the two cases were managed separately. 

#### Criteria used to categorise contacts in South West England (Case 1)

##### Category 1 

Direct contact with case – with symptoms within 21 days: any individual who came into direct contact with the index case and who has developed relevant symptoms associated with exposure to monkeypox within 21 days of contact.

##### Category 2 

Direct contact with case – no symptoms within 21 days: any individual who came into direct contact with the index case who has not developed relevant symptoms within 21 days of contact.

##### Category 3 

Indirect contact with case: any individual with only indirect contact with the index case (see indirect exposure definition below).

A direct contact was defined as any individual who came into direct face-to-face contact with the index case or direct contact with contaminated materials (such as bedding) or sat in the same row or the three rows in front or behind on the flight or shared a taxi. Indirect contact was defined as contact with appropriate personal protective equipment (PPE) or no face-to-face contact and no contact with contaminated materials from the index case.

#### Criteria used to categorise contacts in North West England (Case 2)

##### High-risk exposures 

Direct exposure of broken skin or mucous membranes to a symptomatic (with rash) monkeypox case, their body fluids or potentially infectious material (including on clothing or bedding) without wearing appropriate PPE (including FFP3 or equivalent). This includes: inhalation of respiratory droplets or airborne material from scabs from cleaning rooms where a monkeypox case has stayed, mucosal exposure to splashes and penetrating sharps injury from a used device or through contaminated gloves or clothing. 

##### Intermediate risk exposures 

Intact skin-only contact with a symptomatic (with rash) monkeypox case, their body fluids or potentially infectious material **OR** passengers seated directly next to a case on a plane **OR** people with no direct contact but within 1 m of a symptomatic (with rash) monkeypox case without wearing appropriate PPE (including FFP3 or equivalent). Clinical examination of a monkeypox patient before diagnosis without appropriate PPE (including FFP3 or equivalent). 

##### Low-risk exposures

HCW involved in care of a monkeypox case wearing appropriate PPE (with no known breaches) for all contact episodes **OR** HCW involved in care of a monkeypox case not wearing appropriate PPE for all contact episodes but not within 1 m of the case and with no direct contact with body fluids or potentially infectious material **OR** passengers seated within three rows from the case on a plane, except for passengers sitting directly next to the case **OR** community contacts not within 1 m of the case, i.e. entering the case’s room not wearing PPE without direct contact with the case or his body fluids and maintaining a distance of more than 1 m from the patient. Healthcare staff working in an HCID specialist unit wearing appropriate PPE as described above.

#### Contacts outside the United Kingdom

PHE has contacted public health colleagues in France who identified and contacted passengers who were on the same flight from Lagos, Nigeria to Paris, France as Case 2. The country of residence was provided with details of any non-UK contacts for follow-up where necessary.

### Management of contacts in the United Kingdom

Contacts are being monitored actively or passively depending on their level of exposure risk.

Active surveillance is used for those classified as having a high- or intermediate-risk exposure to a case (direct contact in the South West), their body fluids or potentially infectious materials. This involves the designated PHE contact point contacting the individual every day throughout the 21-day follow-up period to check whether they develop any potential monkeypox prodromal symptoms such as fever, headache, muscle aches, backache, swollen lymph nodes, chills or exhaustion.

Passive surveillance is used for individuals identified as having a low-risk exposure to a case, their body fluids or potentially infectious material (Indirect contact in the South West). They will not be contacted daily during the follow-up period, but will be given a designated PHE contact point to phone if they feel unwell.

Currently 229 of 243 contacts are under investigation; 93 are under active surveillance and 136 are under passive surveillance. Efforts to contact the remainder are ongoing. Following individual risk assessments (see above), 103 of 229 contacts were offered post-exposure prophylaxis (PEP) or pre-exposure prophylaxis (PrEP) with vaccinia vaccine. Fifty-nine community and HCW contacts from the North West were offered PEP (46/59, uptake rate 78%) and 17 community and naval base contacts in the South West were offered PEP (5/17, uptake rate 29%). In addition, 27 HCWs in the HCID units at the Royal Liverpool (Case 2) and the Royal Free hospital (Case 1) managing the patients were offered PrEP. Vaccinees with symptoms consistent with vaccination reactions [2] arising in the 48 h post-vaccination period would be monitored for a further 48 h to discount those in the prodromal phase of monkeypox infection. The individual is advised to discontinue working and self-isolate at home during this time.

Each individual identified as a contact was provided with an information sheet which describes what monkeypox is, how it is spread, and what the symptoms are. This information sheet provides the individual with a designated PHE contact point and telephone number to ring should they develop any symptoms. Contacts can continue to work with no restrictions on their duties if they are asymptomatic. Individuals who develop any symptoms were directed to phone their designated PHE contact point straight away and to stop working until they are assessed by the Imported Fever Service (IFS). Contacts who were planning to travel out of the UK were advised that they may continue with their plans during their 21 days follow-up period if they are asymptomatic. Any contacts under follow-up who are symptomatic are advised not to travel out of the UK. 

## Discussion

Monkeypox is a rare, zoonotic orthopoxvirus with a clinical presentation similar to smallpox [3-5]. The incubation period of monkeypox is usually from 6 to 16 days but can range from 5 to 21 days depending on the route and nature of exposure [6,7]. Initial symptoms typically include fever and lymphadenopathy (a distinctive feature of monkeypox) followed by a maculopapular rash that evolves through different stages [4,5,8,9]. Illness is usually self-limiting and most people recover within several weeks (usually 14 to 21 days), but severe disease can occur in some individuals, including those with underlying conditions such as severe immunosuppression. While evidence of monkeypox infection has been found in a number of animal species in Africa, the natural reservoir host(s) of monkeypox remains unknown [8,9] however, evidence suggests that native African rodents such as rope squirrels (*Funisciurus* spp.), the Gambian pouched rat (*Cricetomys gambianus*) and other rodent species may be potential sources [10-13]. Contact with these animals and consumption of bush meat are thought to be potential methods of zoonotic transmission to humans. Human-to-human transmission is rare but can occur via close contact with skin lesions of an infected person, large respiratory droplets during prolonged face-to-face contact or contaminated objects [5,7].

Since the first human case was recorded in 1970 in the Democratic Republic of Congo (DRC) [14], an increasing number of cases have been reported, suggesting that this is a re-emerging infectious disease [10,15]. In 2018 in Africa, monkeypox cases have been reported from Cameroon, the Central African Republic, the DRC, Liberia and Nigeria. Nigeria has reported a large outbreak of monkeypox that began in September 2017 and peaked in week 41 2017. Since early 2018, between none and five cases have been reported per week. Between September 2017 and 31 August 2018, there were 262 suspected cases across 26 states and 113 confirmed cases, including seven deaths in 16 states [13,16]. The highest number of cases have been reported from the South-South region of Nigeria [16].

Two phylogenetically distinct variants of monkeypox exist, the Central African (Congo-Basin) clade and the West African clade, and these clades differ in disease severity and transmissibility to humans [17]. Of the two clades, the Central African (Congo Basin) clade is associated with more severe disease and transmits more readily by direct contact and large respiratory droplet transmission [6,8,17-19]. On the other hand, the West African clade, found to be responsible for the recent Nigerian outbreak is associated with a milder disease, less mortality and limited human-to-human transmission [20]. Preliminary sequence data for both cases are consistent with the Nigerian strains of the West African clade (data not shown). 

The only other reported cases of human monkeypox infection outside Africa occurred in the United States (US) in 2003 [6,21,22]. They were was traced back to a shipment of West African rodents which were co-housed with pet rodents, including prairie dogs at a pet store. Here we report the first cases of monkeypox infection diagnosed in the UK and Europe and representing importation by a traveller. To our knowledge, this is the only report since the 2003 outbreak in the US and the only report of travel-associated human cases diagnosed outside Africa. 

Currently there is no licensed vaccine specifically for use against monkeypox, but smallpox vaccines are believed to provide a degree of cross-protective immunity against other orthopox viruses, including monkeypox [2,23]. Extensive review of information on a third-generation smallpox vaccine (MVA-BN/Imvanex) [24,25] by the European Medicines Agency concluded that the benefits of this vaccine are greater than its risks and recommended that it be approved for use in the European Union for active immunisation against smallpox in adults [26]. Permissions were obtained from relevant authorities for off-label use of this vaccine in this incident to protect against monkeypox; it is currently being employed for pre- and post-exposure prophylaxis.

The detection of monkeypox cases in non-endemic countries is of public health concern. The diagnosis of two unconnected monkeypox cases within a short time frame in the UK is a highly unusual occurrence and most probably reflects the ongoing monkeypox transmission events in a number of African countries, including Nigeria. This incident reinforces the importance of infectious disease surveillance, clinical awareness and early recognition and isolation, as well as the need to obtain a full travel history for all patients. This incident also highlights the importance of global health security initiatives and the rapid sharing of information, the need for continued collaborations and the strengthening of surveillance systems for emerging and re-emerging infectious diseases globally.
